# Prognostic value of plasma cortisol concentration in dogs with congestive heart failure

**DOI:** 10.1093/jvimsj/aalag063

**Published:** 2026-04-08

**Authors:** Allison K Masters, Jonathan P Mochel, Jiazhang Cai, Andreas Handel, Melissa A Tropf, Melanie J Hezzell, Michael Lu, Mei-Jyun Ciou, Jessica L Ward

**Affiliations:** Department of Veterinary Clinical Sciences, College of Veterinary Medicine, University of Minnesota, St. Paul, MN, United States; SMART Pharmacology Laboratory, Precision One Health Initiative, University of Georgia, Athens, GA, United States; SMART Pharmacology Laboratory, Precision One Health Initiative, University of Georgia, Athens, GA, United States; Department of Epidemiology and Biostatistics, College of Public Health, University of Georgia, Athens, GA, United States; Department of Clinical Sciences, College of Veterinary Medicine, Iowa State University, Ames, IA, United States; Bristol Veterinary School, University of Bristol, Langford House, Langford, Bristol, United Kingdom; Department of Clinical Sciences, College of Veterinary Medicine, Iowa State University, Ames, IA, United States; Department of Clinical Sciences, College of Veterinary Medicine, Iowa State University, Ames, IA, United States; Department of Clinical Sciences, College of Veterinary Medicine, Iowa State University, Ames, IA, United States

**Keywords:** mitral valve disease, mineralocorticoid receptor blocker, renin–angiotensin–aldosterone system, spironolactone

## Abstract

**Background:**

The association of plasma cortisol concentration with prognosis for dogs with congestive heart failure (CHF) is unknown.

**Hypothesis/Objectives:**

To determine whether higher plasma cortisol concentration was independently associated with greater risk of cardiac mortality in dogs with CHF. Additional study aims were to evaluate the associations between other clinical, neurohormonal, and echocardiographic indices and cardiac mortality.

**Animals:**

Thirty-one client-owned dogs with CHF secondary to myxomatous mitral valve disease (MMVD).

**Methods:**

Prospective cohort observational study. Plasma cortisol measurement, urine cortisol-to-creatinine ratio, renal function test results, serum electrolytes, biomarkers of the renin–angiotensin–aldosterone system, N-terminal pro-B-type natriuretic peptide, and echocardiography were performed in dogs with MMVD at first onset of CHF. Plasma cortisol was repeated 7-14 days later. Association between plasma cortisol and other covariates with survival was determined using a proportional hazards regression model.

**Results:**

Plasma cortisol concentration was not associated with cardiac (*P* = .112; hazard ratio [HR] 1.01; 95% CI, 0.998-1.02) or all-cause mortality (*P* = .143; HR 1.01; 95% CI, 0.998-1.02). Treatment with angiotensin-converting enzyme inhibitors (*P* = .021; HR 0.058; 95% CI, 0.0052-0.66) was associated with longer survival to cardiac mortality. Treatment with spironolactone (*P* = .038; HR 0.36; 95% CI, 0.14-0.94), percent fractional shortening (*P* = .034; HR 0.0018; 95% CI, 5.44 × 10^−6^ to 0.61), and lower serum potassium (*P* = .048; HR 2.07; 95% CI, 1.01-4.27) at diagnosis were associated with longer survival to all-cause mortality. Spironolactone treatment at baseline was associated with all-cause mortality on multivariable regression analysis.

**Conclusions and clinical importance:**

Plasma cortisol concentrations were not associated with cardiac mortality in this sample of dogs with CHF.

## Introduction

A major contributor to disease progression in congestive heart failure (CHF) is activation of the renin–angiotensin–aldosterone system (RAAS).^1-5^ The classical RAAS end-product, aldosterone, acts on mineralocorticoid receptors (MRs) to cause cardiovascular effects including vasoconstriction, sodium and water retention, and cardiac fibrosis.^6^ Mineralocorticoid receptor blocker (MRB) drugs, such as spironolactone and eplerenone, are associated with improved clinical outcomes in both humans and dogs with CHF.^7-9^ In disease states, circulating cortisol can bind and activate MRs, similar to aldosterone.^10-12^ Both circulating aldosterone and cortisol concentrations are independent predictors of mortality in CHF in humans, whereas in the subset of people receiving MRBs, cortisol is no longer negatively associated with prognosis.^13^ This suggests that the benefit of MRBs might include blocking the effects of cortisol on MRs, as well as aldosterone.

The role of cortisol in dogs with CHF remains unknown. Based on traditional views of the RAAS, an assumption might be that only dogs with demonstrable circulating RAAS activation would benefit from RAAS blockade using angiotensin-converting enzyme inhibitors (ACEi) or MRBs. However, this is inconsistent with evidence from human clinical trials using MRB therapy in chronic CHF.^7^^,^^8^ These studies demonstrate that MRBs improve prognosis regardless of baseline circulating aldosterone concentration. This suggests that ligands other than aldosterone, such as cortisol, might drive negative cardiovascular outcomes by binding to and activating MRs. Investigation of the relationship between cortisol and prognosis in dogs with CHF could help guide RAAS-modulating treatment recommendations.

The primary aim of this study was to determine whether higher plasma cortisol concentration would be associated with greater risk of cardiac mortality in dogs with CHF secondary to myxomatous mitral valve disease (MMVD). A secondary aim of the study was to determine if other clinical, neurohormonal, and echocardiographic indices would be independently associated with mortality in the same study sample. The primary study hypothesis was that higher plasma cortisol would be independently associated with greater risk of cardiac mortality in dogs with CHF not receiving an MRB. The secondary hypothesis was that biomarkers of RAAS activation would be associated with greater risk of mortality in dogs with CHF, regardless of use of MRBs.

## Materials and methods

Client-owned dogs were prospectively enrolled after diagnosis of left-sided CHF secondary to MMVD at the Iowa State University Lloyd Veterinary Medical Center. This study was approved by the Iowa State University Institutional Animal Care and Use Committee (IACUC protocol no. 19-213 and 22-106). All owners received verbal and written information about the study and gave their consent before inclusion. The sample size was derived from a power calculation based on the hazard ratio for the highest tertile of cortisol to predict all-cause mortality in human CHF studies (HR 2.72; 95% CI, 1.38-5.36).^14^ To achieve α = 0.05 with a power of 80%, an a priori sample size calculation estimated that 19 cardiac death events were required. In order to account for loss to follow-up, and because the median survival time of dogs with MMVD after onset of CHF is approximately 1 year,^15^ an enrollment target of 50 CHF dogs was selected.

### Inclusion and exclusion criteria

Diagnosis of left-sided CHF was based on the radiographic finding of pulmonary edema in conjunction with echocardiographic evidence of severe MMVD.^16^ Dogs were enrolled during their first visit to the hospital for CHF, after emergency stabilization and treatment for CHF. Dogs were excluded if they had received chronic loop diuretic treatment for CHF before the study visit, were receiving glucocorticoid therapy, had previously been diagnosed with hyperadrenocorticism, had severe renal azotemia before diuretic therapy (categorized as suggested by the International Renal Interest Society guidelines as creatinine > 5.0 mg/dL based on a single determination of serum creatinine concentration, with no substaging attempted), or if echocardiography revealed evidence of concurrent congenital or acquired heart disease other than MMVD. Treatment with furosemide for less than 12 h before evaluation at the study center was permitted to allow for stabilization before referral.

### Procedures

Samples were collected at 2 timepoints: on the day of hospital discharge between 13:00 and 14:00 (TP1) and 7 and 14 days after TP1 between 13:00 and 14:00 (TP2). Dogs were fed a moderately sodium-restricted diet (50 and 80 mg/100 kcal) at each timepoint between 07:00 and 08:00. Data were collected at standardized timepoints to minimize variation in cortisol levels and RAAS activity related to individual circadian rhythm, feeding time, and diet.^17-19^ Ethylenediaminetetraacetic acid (EDTA) plasma cortisol concentrations were measured at 2 timepoints to control for the effect of hospitalization on cortisol and to measure circulating cortisol levels in dogs with CHF after initial stabilization. Treatment for CHF was based on the individual dog according to the standard of care for MMVD stage C, which changed during the study period,^16^^,^^20^ and was at the discretion of the attending clinician. Owners were provided with a medication log to track administration of all medications to their dog.

Data collected at TP1 included: Doppler blood pressure, complete echocardiogram, free plasma cortisol, bound plasma cortisol, urine cortisol-to-creatinine ratio, serum RAAS biomarker concentrations, renal chemistry panel, N-terminal pro-B-type natriuretic peptide (NT-proBNP), and C-reactive protein (CRP). Doppler blood pressure measurement and the complete echocardiogram were not exclusively performed during the 13:00-14:00 sampling window at TP1. Data collected at TP2 included: completed medication tracking log, free plasma cortisol, and bound plasma cortisol.

### Echocardiography and Doppler blood pressure

All echocardiographic examinations were performed and assessed by a board-certified cardiologist or supervised resident. Echocardiography was performed using simultaneous ECG monitoring with an ultrasound unit using 5-12 MHz phased-array transducers (EPIQ 7, Philips Ultrasound, Bothell, WA, USA). Transthoracic 2-dimensional, M-mode, and Doppler echocardiography was performed in right and left lateral recumbency in each dog while unsedated.^21^^,^^22^ Measurements recorded included: 2-dimensional left atrium-to-aorta ratio (LA:Ao) from the right parasternal short-axis basilar view, short-axis M-mode left ventricular internal diameter in diastole normalized to body-weight (LVIDdN),^23^ short-axis M-mode left ventricular fractional shortening (LV FS%), and E wave velocity from the left apical view. All echocardiographic measurements were made on 3 consecutive cardiac cycles, and the mean value was used for statistical analyses. Noninvasive systemic arterial blood pressure was obtained using Doppler with gentle restraint in each dog following standard methods.^24^

### Plasma cortisol analysis

Three milliliter EDTA tubes were centrifuged within 30 min at room temperature at 1500×*g* for 30 min and the resulting plasma was transferred into cryovials stored at −80°C. Batch analysis of free plasma cortisol and bound plasma cortisol were performed by the Iowa State University Analytical Chemistry Laboratory using rapid equilibrium dialysis followed by liquid chromatography–tandem mass spectrometry in which cortisol-d4 was used as a surrogate analyte for calibration. Equilibrium dialysis was performed for 300 μL of each plasma sample dialyzed against 550 μL of phosphate-buffered saline solution. Plasma samples were prepared by protein precipitation and buffer samples were prepared by liquid–liquid extraction before liquid chromatography–tandem mass spectrometry analysis. The lower limit of detection of free plasma cortisol on the assay utilized was 4.7 ng/mL. Samples with a free plasma cortisol of < 4.7 ng/mL were assigned a value of 4.7 ng/mL for statistical analysis. Samples in which a free plasma cortisol could not be detected were assigned a value of 0 ng/mL for statistical analysis. Total plasma cortisol was calculated as the sum of free and bound plasma cortisol.

### RAAS biomarker analysis

Five milliliter additive-free tubes were centrifuged at room temperature at 1500×*g* for 30 min and the resulting serum was transferred into cryovials and stored at −80°C. Batched serum samples were shipped frozen on dry ice to an academic service (University of Kentucky RAAS Lab, Lexington, KY) for serum RAAS biomarker analysis. RAAS hormones quantified in the assay include: angiotensin I (AngI), angiotensin II (AngII), angiotensin III (AngIII), angiotensin IV (AngIV), angiotensin 1-7 (Ang1-7), angiotensin 1-5 (Ang1-5), and aldosterone. Concentrations of RAAS analytes at equilibrium were quantified via liquid chromatography-mass spectrometry at the University of Kentucky RAAS Lab (Lexington, KY), using previously validated and described methods (ie, RAS Fingerprint, developed by Attoquant Diagnostics GmBH, Vienna, Austria).^25-28^ Briefly, serum samples that did not contain a protease inhibitor were subjected to ex vivo incubation at 37°C for 1 h to generate stable equilibrium. The equilibrated serum samples were then subsequently stabilized through the addition of an enzyme inhibitor cocktail, and spiked with stable isotope labeled internal standards for each angiotensin metabolite as well as with the deuterated internal standard for aldosterone (aldosterone D4) at a concentration of 200 pg/mL. The samples then underwent C-18-based solid-phase-extraction and were subjected to liquid chromatography-mass spectrometry analysis using a reversed-phase analytical column (Acquity UPLC C18, Waters) operating in line with a Xevo TQ-S triple quadrupole mass spectrometer (Waters Xevo TQ/S, Milford, MA) in multiple reaction monitoring mode. Internal standards were used to correct for analyte recovery across the sample preparation procedure in each individual sample. Quantification was performed using software (MassLynx/Target/Lynx, Waters) via integration of the total ion chromatogram obtained from a sum of quantifier transitions that have been optimized for sensitivity and specificity. Analyte concentrations were reported in pM and calculated considering the corresponding response factors determined in appropriate calibration curves in sample matrix, when integrated signals exceeded a signal-to-noise ratio of 10. The lower limit of quantification was 3.0 pM/L for AngI, 2.0 pM/L for AngII, 3.0 pM/L for Ang1-7, 2.0 pM/L for Ang1-5, 3.0 pM/L for AngIII, 2.0 pM/L for AngIV, and 15 pM/L for aldosterone.

The following surrogate markers were calculated based on serum RAAS biomarker concentrations: plasma renin activity (PRA-S), ACE activity (ACE-S), and adrenal responsiveness (AA2-ratio). PRA-S was calculated as the sum of AngI + AngII. ACE-S was calculated by dividing AngII/AngI. AA2, a measure of adrenal responsiveness to angiotensin II, was calculated as aldosterone/AngII. All RAAS analytes and PRA-S are reported in pM/L. AA2 and ACE-S are ratios of pM/L:pM/L.

### Other biochemical and urine testing

Serum samples were analyzed on the day of sampling for a renal chemistry panel (creatinine, blood urea nitrogen [BUN], sodium, potassium, chloride, bicarbonate, calcium, phosphorus, and albumin) by the Iowa State University Clinical Pathology Laboratory at TP1. Banked cryopreserved plasma, serum, and urine samples from TP1 were shipped to a commercial laboratory (IDEXX BioAnalytics. Westbrook, ME, USA) for batched analysis of plasma NT-proBNP, serum CRP, and urine cortisol-to-creatinine ratio.

### Outcome

Owners or referring veterinarians were contacted by telephone or e-mail at the end of the 2-year study period to determine long-term outcome, including date and cause of death. The primary outcome measure evaluated was cardiac mortality, defined as death or euthanasia due to progressive CHF or sudden cardiac death as documented in the medical records when available or by direct owner or referring veterinarian communication. All-cause mortality, defined as death or euthanasia during the study period due to any cause, was also evaluated. Survival time was calculated as the number of days from TP1 to the date of death or euthanasia.

### Statistical analysis

All statistical analyses were performed in R (version 4.5.1). Data preprocessing and analysis were conducted using the packages tidyverse, readxl, PerformanceAnalytics, car, and survival. Continuous variables were summarized as median with interquartile range, and minimum–maximum values. Summary statistics for all variables were generated programmatically and exported for reporting.

Survival time was defined as the number of days from TP1 to death. Because all dogs in the cohort experienced the event of interest (death), the survival outcome was not censored. A constant event indicator of 1 was therefore used in all Cox proportional hazards models. Two subsets of data were analyzed: (1) all-cause mortality, including all dogs, and (2) cardiac mortality, restricted to dogs with cardiac death.

Univariate Cox proportional hazards models were used to evaluate the association between each variable and survival time. For each model, the hazard ratio, 95% CI, and the corresponding *P*-value were calculated. Because multiple variables were tested, false discovery rate (FDR)-adjusted *P*-values were computed using the Benjamini–Hochberg method to account for multiple comparisons. We report both the original (unadjusted) and FDR-adjusted *P*-values for transparency, but primary inferences were guided by the unadjusted *P*-values to avoid overlooking potentially meaningful biological signals that warrant further investigation in larger cohorts. Multivariable Cox regression models were constructed for cardiac and all-cause mortality using total plasma cortisol at TP1, spironolactone treatment at TP1, and age on unadjusted univariable analysis for cardiac and all-cause mortality.

Kaplan–Meier survival curves were generated for categorical variables significantly associated with survival in the univariable Cox proportional hazards analysis and total plasma cortisol concentrations at TP1 (separated by tertile). Curves were compared descriptively to illustrate trends in survival distribution.

## Results

Thirty-one dogs with first-time CHF secondary to MMVD were enrolled between August 2019 and March 2021. One dog was excluded for oral administration of corticosteroid at the time of enrollment. Landmark analysis using a predefined landmark time of 14 days after the diagnosis of CHF was performed to mitigate potential bias from early deaths. Dogs that died or were censored before this time were excluded from the analysis (*n* = 4). Survival time was recalculated from the 14-day landmark onward, and Cox proportional hazards models were applied to evaluate the association between treatment and survival beyond that point to focus on longer-term survival while minimizing the confounding influence of early death events (Figure S1). Median age of the remaining 26 dogs was 11 years (range 7-15 years). Twelve were castrated males and 14 were spayed females. Breeds included mixed breed (*n* = 9), Cavalier King Charles Spaniel (*n* = 3), Dachshund (*n* = 2), Chihuahua (*n* = 2), Shih Tzu (*n* = 2), and one each of Basset Hound, Boston Terrier, corgi, Italian Greyhound, Maltese, Miniature Schnauzer, Pomeranian, and Rat Terrier. Median body weight of the CHF dogs was 6.9 kg (range 1.7-23.6 kg). Medications prescribed at TP1 and TP2 are detailed in Table 1. All 26 CHF dogs were prescribed furosemide at TP1. Echocardiography at TP1 confirmed severe MMVD in all 31 CHF dogs (Table 2).

**Table 1:** Number of dogs with congestive heart failure secondary to myxomatous mitral valve disease prescribed cardiac medications at time point 1 (TP1) and at time point 2 (TP2), 7-14 days later.

Medication	Number of dogs at TP1 (total = 26)	Number of dogs at TP2 (total = 26)	Dosage range at TP1 (mg/kg/day)	Dosage range at TP2 (mg/kg/day)
**Furosemide**	26	26	2.7-4.8	2.1-4.8
**Pimobendan**	26	26	0.46-0.85	0.46-1.1
**Enalapril**	17	17	0.5-1.2	0.37-1.2
**Benazepril**	4	6	0.42-1.2	0.42-1.2
**Spironolactone**	7	24	1.6-2.7	1.3-4.3
**Amlodipine**	1	2	0.15	0.14-0.15
**Potassium gluconate**	3	3		

**Table 2:** Clinicopathologic and echocardiographic variables in dogs with congestive heart failure secondary to myxomatous mitral valve disease (*n* = 26) at time point 1. Data are reported as median (interquartile range) and range (number of samples).

	Median (interquartile range)	Range (number of samples)
**Systolic arterial blood pressure measured by Doppler (mmHg)**	129 (115.8-140)	80-190 (*n* = 26)
**Heart rate (beats/minute)**	150 (131.3-168)	90-200 (*n* = 26)
**LA:Ao**	2.0 (1.8-2.4)	1.4-3.4 (*n* = 26)
**LVIDdN (cm)**	2.0 (1.8-2.1)	1.2-2.6 (*n* = 26)
**LV FS%**	53.9 (46.6-58.7)	38.2-64.9 (*n* = 26)
**E wave velocity (m/s)**	1.1 (0.8-1.3)	0.68-1.84 (*n* = 23)
**Sodium (mEq/L)**	142.5 (140-144)	134-154 (*n* = 26)
**Potassium (mEq/L)**	4.0 (3.7-4.6)	2.9-5.0 (*n* = 26)
**Chloride (mEq/L)**	108 (103.3-110.8)	92-120 (*n* = 26)
**Bicarbonate (mEq/L)**	24.0 (23.0-27.0)	17-36 (*n* = 26)
**Calcium (mg/dL)**	10.4 (10.0-10.7)	9.1-11.6 (*n* = 26)
**Phosphorus (mg/dL)**	5.7 (4.5-6.4)	3.7-8.2 (*n* = 26)
**BUN (mg/dL)**	24.5 (18.0-39.5)	9-66 (*n* = 26)
**Creatinine (mg/dL)**	1.2 (0.9-1.3)	0.5-1.7 (*n* = 26)
**Albumin (mg/dL)**	3.6 (3.1-3.8)	2.9-4.8 (*n* = 26)
**Anion gap**	14.0 (11.0-17.8)	7-23 (*n* = 26)

Abbreviations: BUN = blood urea nitrogen; LA:Ao = left-atrium-to-aorta ratio; LV FS% = left ventricular fractional shortening; LVIDdN = left-ventricular internal diameter in diastole normalized to body-weight

### Plasma cortisol analysis

Descriptive statistics for plasma cortisol concentration in dogs with CHF are summarized in Table 3. Free, bound, and total plasma cortisol were assessed in all dogs at TP1 and in 23 dogs at TP2. Four dogs had measurable free plasma cortisol at TP1 and 10 dogs had measurable free plasma cortisol at TP2; the remaining dogs had free plasma cortisol of < 4.7 ng/mL (ie, below the lower limit of quantification of the assay). Due to the frequency of missing data for measurable free plasma cortisol in the dogs studied, this analyte was excluded from the analysis of intraindividual cortisol variability. No significant difference in intraindividual bound or total plasma cortisol between TP1 and TP2 was detected in the CHF dogs (*P* = .88 and .83, respectively).

**Table 3:** Neurohormonal variables in dogs with congestive heart failure secondary to myxomatous mitral valve disease (*n* = 26).

	Median (interquartile range)	Range (number of samples)
**Urine cortisol-to-creatinine ratio**	86.5 (60.2-120.3)	34.0-196.7 (*n* = 24)
**NT-proBNP (pM/L)**	1838 (1127-2549)	452-3460 (*n* = 23)
**CRP (mg/dL)**	8.7 (2.7-13.3)	<0.8-26.7 (*n* = 22)
**Free plasma cortisol TP1 (ng/mL)**	4.7 (4.7-4.7)	0-22.7 (*n* = 26)
**Bound plasma cortisol TP1 (ng/mL)**	48.4 (36.6-71.9)	4.7-207 (*n* = 26)
**Total plasma cortisol TP1 (ng/mL)**	53.2 (41.3-76.6)	9.4-213.7 (*n* = 26)
**Free plasma cortisol TP2 (ng/mL)**	4.7 (0-7.8)	0-36.3 (*n* = 20)
**Bound plasma cortisol TP2 (ng/mL)**	50.3 (32.8-102.3)	12.2-233 (*n* = 20)
**Total plasma cortisol TP2 (ng/mL)**	49.7 (32.1-94.8)	12.2-251 (*n* = 20)
**AngI (pM/L)**	908.2 (682.9-1304.8)	11.6-2794.1 (*n* = 26)
**AngII (pM/L)**	178.9 (44.9-377.3)	15.4-1175.7 (*n* = 26)
**AngIII (pM/L)**	33.3 (6.0-77.5)	1.5-395.2 (*n* = 26)
**AngIV (pM/L)**	46.8 (9.6-84.5)	1.0-447.8 (*n* = 26)
**Ang1-7 (pM/L)**	346.5 (220.0-435.4)	1.5-866.1 (*n* = 26)
**Ang1-5 (pM/L)**	241.5 (50.7-396.3)	1.0-1608.8 (*n* = 26)
**Aldosterone (pM/L)**	203.1 (68.9-545.6)	7.5-1303.2 (*n* = 26)
**PRA-S (pM/L)**	1121.8 (880.0-1702.1)	31.6-3346 (*n* = 26)
**ACE-S (pM/L:pM/L)**	0.3 (0.1-0.5)	0.02-1.7 (*n* = 26)
**AA2 (pM/L:pM/L)**	1.2 (0.6-1.8)	0.1-8.8 (*n* = 26)

Data were collected at time point 1 (TP1) when not specified. Data obtained at time point 2 (TP2) were collected 7-14 days later. Data are reported as median (interquartile range) and range (number of samples).

Abbreviations: AA2 = ratio of aldosterone to angiotensin II; ACE-S = ratio of angiotensin II to angiotensin I; Ang = angiotensin; CRP = C-reactive protein; NT-proBNP = N-terminal pro-B-type natriuretic peptide; PRA-S = sum of angiotensin I and angiotensin II.

### RAAS biomarkers

Descriptive statistics for RAAS analytes in dogs with CHF are summarized in Table 3.

### Cardiac mortality

**Figure 1:**
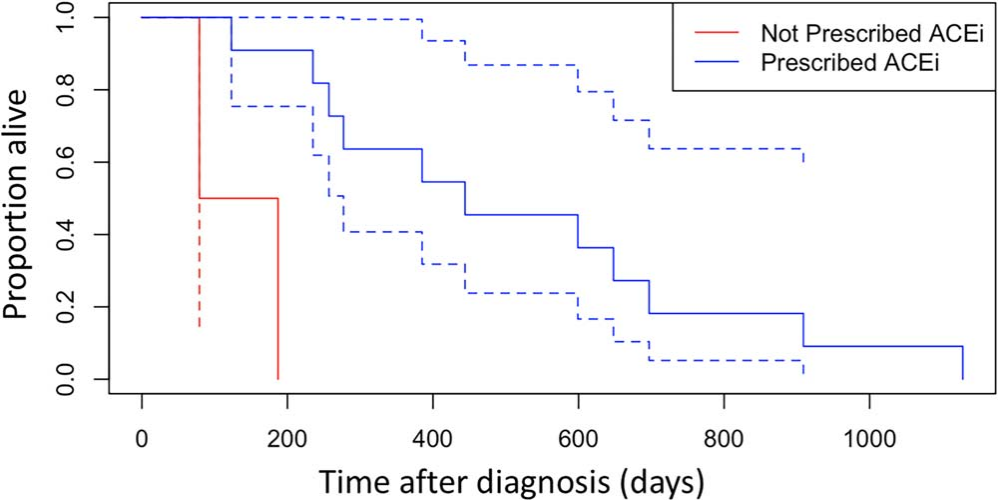
Kaplan–Meier curve demonstrating median cardiac survival time for dogs with congestive heart failure secondary to myxomatous mitral valve disease that were prescribed an angiotensin-converting enzyme inhibitor at TP1 (blue) and those that were not prescribed an angiotensin-converting enzyme inhibitor at TP1 (red). The dashed lines represent the 95% CIs for the survival curves. Cases with survival time of less than 14 days were removed before generation of Kaplan–Meier curve.

**Figure 2:**
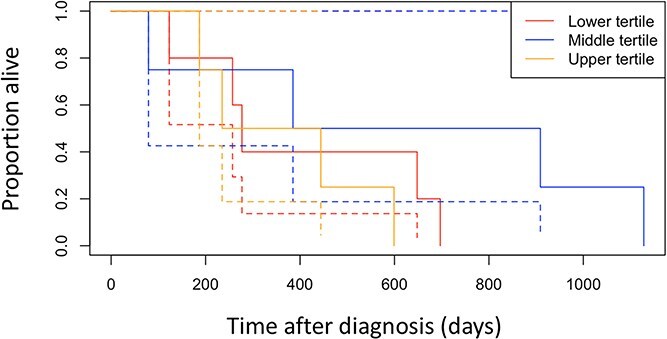
Kaplan–Meier curve demonstrating median cardiac survival time for dogs with congestive heart failure secondary to myxomatous mitral valve disease based on total plasma cortisol at TP1 tertiles. The dashed lines represent the 95% CIs for the survival curves. Cases with survival time of less than 14 days were removed before generation of Kaplan–Meier curve.

Thirteen dogs experienced cardiac death with a median survival time of 371 days (range 65-1144 days). Results of the cardiac mortality univariate analysis are detailed in Table 4. Time to cardiac death was associated with ACEi treatment (*P* = .021) in univariable analysis. Median survival time for CHF dogs experiencing cardiac death treated with ACEi (*n* = 11) was 430 days (range 109-1114 days), vs 119 days (range 65-173 days) for those not treated with ACEi (*n* = 2; Figure 1). Time to cardiac death was not associated with free, bound, or total plasma cortisol at TP1 (*P* = .55, .10, and .11, respectively) or TP2 (*P* = .70, .74, and .73, respectively; Figure 2). No other clinical, echocardiographic, or clinicopathologic covariates were associated with cardiac death on univariable analysis. No variables were predictive of cardiac mortality in a multivariable model including plasma cortisol at TP1, spironolactone treatment at TP1, and age (Table 5).

**Table 4:** Relationship between clinical, neurohormonal, and echocardiographic variables in dogs with congestive heart failure secondary to myxomatous mitral valve disease and cardiac mortality.

	Hazard ratio (95% CI)	*P*-value (adjusted *P*-value)
**Sex (male castrated)**	2.44 (0.730-8.18)	.147 (.720)
**Age (years)**	0.696 (0.449-1.08)	.107 (.720)
**Weight (kg)**	1.01 (0.891-1.15)	.853 (.878)
**Systolic arterial blood pressure measured by Doppler (mmHg)**	1.01 (0.987-1.03)	.502 (.776)
**Heart rate (beats/minute)**	1.01 (0.985-1.03)	.564 (.778)
**LA:Ao**	1.93 (0.670-5.54)	.224 (.720)
**LVIDdN (cm)**	5.57 (0.778-39.9)	.0872 (.720)
**LV FS%**	0.00118 (3.27 × 10^−8^-42.4)	.208 (.720)
**E wave velocity (m/s)**	8.13 (0.686-96.4)	.0966 (.720)
**Sodium (mEq/L)**	0.869 (0.700-1.08)	.205 (.720)
**Potassium (mEq/L)**	1.47 (0.558-3.88)	.435 (.763)
**Chloride (mEq/L)**	0.983 (0.873-1.11)	.775 (.861)
**Bicarbonate (mEq/L)**	0.964 (0.853-1.09)	.560 (.778)
**Calcium (mg/dL)**	0.604 (0.278-1.31)	.203 (.720)
**Phosphorus (mg/dL)**	0.723 (0.445-1.17)	.190 (.720)
**BUN (mg/dL)**	0.984 (0.926-1.04)	.589 (.786)
**Creatinine (mg/dL)**	0.406 (0.0675-2.44)	.325 (.720)
**Albumin (g/dL)**	2.20 (0.429-11.2)	.345 (.720)
**Anion gap**	1.02 (0.864-1.21)	.796 (.861)
**Urine cortisol-to-creatinine ratio**	1.01 (0.990-1.03)	.360 (.720)
**NT-proBNP (pM/L)**	1.00 (0.999-1.00)	.269 (.720)
**C-reactive protein (mg/dL)**	1.01 (0.935-1.08)	.856 (.878)
**Free plasma cortisol TP1**	1.04 (0.914-1.18)	.552 (.778)
**Bound plasma cortisol TP1**	1.01 (0.998-1.02)	.103 (.720)
**Total plasma cortisol TP1**	1.01 (0.998-1.02)	.112 (.720)
**Free plasma cortisol TP2**	0.969 (0.827-1.14)	.701 (.848)
**Bound plasma cortisol TP2**	0.997 (0.978-1.01)	.742 (.848)
**Total plasma cortisol TP2**	0.997 (0.981-1.01)	.733 (.848)
**AngI (pM/L)**	0.999 (0.997-1.00)	.305 (.720)
**AngII (pM/L)**	1.00 (0.998-1.00)	.973 (.973)
**AngIII (pM/L)**	1.01 (0.995-1.02)	.258 (.720)
**AngIV (pM/L)**	1.00 (0.992-1.02)	.458 (.763)
**Ang1-7 (pM/L)**	1.00 (0.997-1.01)	.646 (.834)
**Ang1-5 (pM/L)**	1.00 (0.99-1.00)	.257 (.720)
**Aldosterone (pM/L)**	1.00 (0.999-1.00)	.309 (.720)
**PRA-S (pM/L)**	1.00 (0.998-1.00)	.539 (.778)
**ACE-S (pM/L:pM/L)**	1.61 (0.165-15.6)	.683 (.848)
**AA2 (pM/L:pM/L)**	1.20 (0.756-1.89)	.445 (.763)
**Treatment with ACEi**	0.0581 (0.00515-0.656)	.021 (.720)
**Treatment with spironolactone TP1**	0.593 (0.153-2.30)	.450 (.763)

Data were collected at time point 1 (TP1) when not specified. Data obtained at time point 2 (TP2) were collected 7-14 days later. *P*-values, hazard ratios, and CIs determined by univariate Cox proportional hazards regression model. False discovery rate-adjusted *P*-values were computed using the Benjamini–Hochberg method to account for multiple comparisons.

Abbreviations: AA2 = ratio of aldosterone to angiotensin II; ACEi = angiotensin-converting enzyme inhibitor; ACE-S = ratio of angiotensin II to angiotensin I; Ang = angiotensin; BUN = blood urea nitrogen; LA:Ao = left-atrium-to-aorta ratio; LV FS% = left ventricular fractional shortening; LVIDdN = left-ventricular internal diameter in diastole normalized to body-weight; NT-proBNP = N-terminal pro-B-type natriuretic peptide; PRA-S, sum of angiotensin I and angiotensin II.

**Table 5:** Hazard ratios for cardiac mortality in dogs with congestive heart failure secondary to myxomatous mitral valve disease using total plasma cortisol at TP1, spironolactone treatment at TP1, and age in the model. *P*-values, hazard ratios, and CIs determined by multivariable cox proportional hazards regression model.

	Cardiac mortality hazard ratio	Cardiac mortality CI	Cardiac mortality *P*-value
**Total plasma cortisol TP1**	1.01	1.00-1.02	.18
**Spironolactone treatment TP1**	0.50	0.12-2.04	.34
**Age (years)**	0.71	0.44-1.13	.14

### All-cause mortality

Twenty-six dogs died during the study period with a median all-cause survival time of 334 days (40-1209 days). Results of the all-cause mortality univariate analysis are detailed in Table 6. Time to all-cause mortality was not associated with free, bound, or total plasma cortisol at TP1 (*P* = .42, .14, and .14, respectively) or TP2 (*P* = .82, .83, and .75, respectively). Survival time to all-cause mortality was associated with larger LV FS% (*P* = .034), spironolactone treatment at TP1 (*P* = .038), and lower serum potassium concentration (*P* = .048) on univariate analysis. All-cause mortality was not associated with any other covariates. All-cause mortality remained associated with spironolactone treatment at TP1 (*P* = .04) in a multivariable analysis, including total plasma cortisol at TP1, spironolactone treatment at TP1, and age (Table 7).

**Table 6:** Relationship between clinical, neurohormonal, and echocardiographic variables in dogs with congestive heart failure secondary to myxomatous mitral valve disease and all-cause mortality.

	Hazard ratio (95% CI)	*P*-value (adjusted P-value)
**Sex (male castrated)**	1.62 (0.724-3.62)	.241 (.599)
**Age (years)**	0.855 (0.680-1.07)	.179 (.598)
**Weight (kg)**	1.03 (0.943-1.13)	.495 (.729)
**Systolic arterial blood pressure measured by Doppler (mmHg)**	1.00 (0.988-1.02)	.631 (.765)
**Heart rate (beats/minute)**	1.00 (0.991-1.02)	.523 (.730)
**LA:Ao**	1.31 (0.645-2.66)	.455 (.730)
**LVIDdN (cm)**	2.52 (0.769-8.24)	.127 (.520)
**LV FS%**	0.00182 (5.44 × 10^−6^-0.612)	.034 (.520)
**E wave velocity (m/s)**	3.38 (0.897-12.7)	.072 (.520)
**Sodium (mEq/L)**	1.00 (0.891-1.13)	.977 (.877)
**Potassium (mEq/L)**	2.07 (1.01-4.27)	.048 (.520)
**Chloride (mEq/L)**	1.05 (0.988-1.12)	.114 (.520)
**Bicarbonate (mEq/L)**	0.950 (0.867-1.04)	.263 (.599)
**Calcium (mg/dL)**	0.857 (0.514-1.43)	.552 (.730)
**Phosphorus (mg/dL)**	0.788 (0.543-1.14)	.211 (.599)
**BUN (mg/dL)**	0.991 (0.963-1.02)	.565 (.730)
**Creatinine (mg/dL)**	0.665 (0.175-2.53)	.549 (.730)
**Albumin (g/dL)**	0.713 (0.307-1.66)	.431 (.730)
**Anion gap**	0.930 (0.827-1.05)	.223 (.599)
**Urine cortisol-to-creatinine ratio**	0.995 (0.985-1.00)	.265 (.599)
**NT-proBNP (pM/L)**	1.00 (0.999-1.00)	.270 (.599)
**C-reactive protein (mg/dL)**	1.00 (1.00-1.00)	.875 (.921)
**Free plasma cortisol TP1**	1.04 (0.949-1.13)	.418 (.730)
**Bound plasma cortisol TP1**	1.01 (0.998-1.02)	.135 (.520)
**Total plasma cortisol TP1**	1.01 (0.998-1.02)	.143 (.520)
**Free plasma cortisol TP2**	1.01 (0.954-1.06)	.819 (.896)
**Bound plasma cortisol TP2**	0.999 (0.991-1.01)	.829 (.896)
**Total plasma cortisol TP2**	0.998 (0.989-1.01)	.753 (.886)
**AngI (pM/L)**	0.999 (0.998-1.00)	.094 (.520)
**AngII (pM/L)**	0.999 (0.998-1.00)	.489 (.730)
**AngIII (pM/L)**	0.999 (0.994-1.00)	.797 (.896)
**AngIV (pM/L)**	0.999 (0.994-1.00)	.603 (.754)
**Ang1-7 (pM/L)**	0.999 (0.997-1.00)	.302 (.605)
**Ang1-5 (pM/L)**	1.00 (0.999-1.00)	.443 (.730)
**Aldosterone (pM/L)**	1.00 (0.999-1.00)	.944 (.968)
**PRA-S (pM/L)**	1.00 (0.999-1.00)	.139 (.520)
**ACE-S (pM/L:pM/L)**	1.73 (0.633-4.74)	.284 (.599)
**AA2 (pM/L:pM/L)**	1.08 (0.861-1.36)	.500 (.730)
**Treatment with ACEi**	0.289 (0.078-1.06)	.061 (.520)
**Treatment with spironolactone TP1**	0.361 (0.138-0.943)	.038 (.520)

Data were collected at time point 1 (TP1) when not specified. Data obtained at time point 2 (TP2) were collected 7-14 days later. *P*-values, hazard ratios, and CIs determined by univariate Cox proportional hazards regression model. False discovery rate-adjusted *P*-values were computed using the Benjamini–Hochberg method to account for multiple comparisons.

Abbreviations: AA2 = ratio of aldosterone to angiotensin II; ACEi = angiotensin-converting enzyme inhibitor; ACE-S = ratio of angiotensin II to angiotensin I; Ang = angiotensin; BUN = blood urea nitrogen; LA:Ao = left-atrium-to-aorta ratio; LV FS% = left ventricular fractional shortening; LVIDdN = left-ventricular internal diameter in diastole normalized to body-weight; NT-proBNP = N-terminal pro-B-type natriuretic peptide; PRA-S = sum of angiotensin I and angiotensin II.

**Table 7:** Hazard ratios for all-cause mortality in dogs with congestive heart failure secondary to myxomatous mitral valve disease using total plasma cortisol at TP1, spironolactone treatment at TP1, and age in the model.

	All-cause mortality hazard ratio	All-cause mortality CI	All-cause mortality *P*-value
**Total plasma cortisol TP1**	1.01	1.00-1.02	.07
**Spironolactone treatment TP1**	0.36	0.14-0.95	.04
**Age (years)**	0.88	0.71-1.09	.24

*P*-values, hazard ratios, and CIs determined by multivariable Cox proportional hazards regression model.

## Discussion

In this cohort of dogs with CHF, plasma cortisol concentrations were not associated with cardiac or all-cause mortality, contrary to the primary study hypothesis. Of the clinical and clinicopathologic variables tested in this study, only treatment with ACEi was associated with longer time to cardiac death. Treatment with spironolactone, serum potassium concentration, and LV FS% were associated with longer time to all-cause mortality. Treatment with spironolactone at TP1 remained significant in the multivariate analysis for all-cause mortality. Concentrations of individual RAAS metabolites and NT-proBNP were not associated with outcome in this study sample.

Treatment of dogs with CHF secondary to MMVD using RAAS-modulating drugs, such as ACEi and MRBs, is standard practice.^16^ Yet, studies conflict on the positive effect of these drugs on survival in dogs with CHF. Some studies demonstrate improved survival in dogs with CHF^9^^,^^29-33^ while others do not.^34^ Although this study was not designed to investigate the effects of RAAS-modulating drugs on survival, ACEi and MRB treatments were both positively associated with survival times on univariate analysis despite there being no relationship between circulating RAAS analyte concentrations and survival. The association between ACEi treatment and death was not present on multivariate analysis and might represent a false positive due to multiple comparisons. This is supported by the lack of significance of ACEi treatment in the univariate analysis after FDR correction (*P* = .720). In human clinical trials using MRB therapy in chronic CHF,^7^^,^^8^ MRB use is associated with improved prognosis, regardless of initial circulating aldosterone level. This might reflect high variability in circulating aldosterone, or could potentially suggest a role for other MR ligands, such as cortisol, in exacerbating cardiovascular disease.

Cortisol binds MRs with the same affinity as aldosterone, and circulating concentrations of free cortisol are typically 100-200 times higher than aldosterone.^35^^,^^36^ In human heart failure patients, elevated circulating cortisol is an independent predictor of cardiac mortality.^13^^,^^37^ This is suspected to be due to the ability of cortisol to bind to cardiac MRs in disease states. These actions are beneficial in the short-term, but lead to negative pathophysiologic consequences in chronic CHF, including increased preload and afterload as well as pathologic cardiac hypertrophy and fibrosis.^6^

In contrast to the findings reported in human patients, circulating plasma cortisol was not associated at any timepoint with cardiac mortality or all-cause mortality in this cohort of dogs with CHF. One explanation for this finding is the failure to reach the study enrollment target, leading to only 13 cardiac deaths in the cohort. As a result, the study is underpowered and might have resulted in type II error. Another explanation is the widespread use of spironolactone in dogs enrolled in this study. This study was planned before the publication of updated ACVIM consensus guidelines for the diagnosis and treatment of MMVD in dogs in 2019, which recommends initiation of therapy with spironolactone in all dogs with CHF secondary to MMVD,^16^ and before publication of the BESST study, which demonstrates a survival benefit of spironolactone when added to furosemide and ACEi treatment in CHF secondary to MMVD.^33^ Based on this evidence, prescribing practices at our hospitals changed such that the majority of dogs in this study (*n* = 24) were prescribed spironolactone at TP1 (*n* = 7) or TP2 (*n* = 17), which did not allow sufficient sample size to assess the relationship between plasma cortisol and cardiac death in dogs with CHF not receiving MRB treatment.

The lack of association between circulating RAAS analytes and mortality in our study sample might be explained by the use of RAAS-modulating drugs in the dogs studied. Twenty-three dogs were treated with ACEi (17 enalapril, 6 benazepril) and 24 were treated with spironolactone. Only one dog did not receive at least one RAAS-modulating drug during the study period due to concern for side effects. The dog survived 329 days and experienced non-cardiac death. Despite a lack of association between circulating RAAS analytes and survival, the dogs with CHF in this study had global RAAS upregulation at TP1. Specifically, median AngI, AngIII, AngIV, aldosterone, Ang1-7, and PRA-S at TP1 in dogs with CHF were all above the established normal ranges for healthy dogs.^38^^,^^39^ This is likely due to a combination of severe cardiovascular disease and prior furosemide treatment.^40^^,^^41^ This global RAAS upregulation could support continued use of broad RAAS inhibition in dogs with CHF secondary to MMVD.

Hypokalemia is negatively associated with survival times in both dogs with CHF secondary to MMVD^42^ and human patients with chronic CHF.^43^^,^^44^ However, in the present study, higher serum potassium concentration was associated with higher risk of all-cause mortality. Given that the highest potassium in the CHF dogs in this study was 5.0 mEq/L, the negative cardiovascular effects of hyperkalemia cannot explain this finding. At the time of sampling, CHF dogs had been receiving loop diuretic treatment. It is possible the more aggressive diuresis in the acute setting resulted in both lower potassium and improved survival. The association between furosemide dose administered during the initial CHF episode and survival was not directly assessed in the present study. In addition, only 5 dogs in this study were hypokalemic (serum potassium < 3.5 mEq/L) and the remaining 25 dogs were normokalemic. The low number of hypokalemic dogs in the study could explain why lower serum potassium concentration was not associated with reduced survival time.

The limitations of this study include the small sample size, which fell below our initial target enrollment but still provided adequate numbers of dogs that died based on our original sample size calculation to assess the effect of cortisol on all-cause mortality. However, the study might have been underpowered to evaluate alternative covariates or outcomes, including cardiac mortality. Due to limited sample size and the large number of variables tested, no variables remained significant after FDR correction. The effects of cardiac medications administered before enrollment were not analyzed and might have affected study results. Changes in evidence-based treatment recommendations during the study period resulted in few dogs being managed without MRB treatment, which had been the original target sample for evaluation of the effect of cortisol on survival. Treatment of dyspnea-associated anxiety with narcotics and anxiolytics was not standardized nor analyzed in this study and could have affected the study results, specifically by reducing cortisol and RAAS biomarkers. Treatment in this study was not strictly standardized, such that clinician biases in prescribing practices might have resulted in dogs with perceived poor survival not receiving RAAS-mitigating drugs. In addition, because treatments were not randomized, factors that might have influenced survival times could have changed clinicians’ treatment decisions. Cortisol and RAAS biomarkers were assessed after initial emergency stabilization and treatment of CHF rather than at original presentation. This timing was chosen for consistency with previous studies of cortisol in human patients with CHF^13^ and to minimize the effect of dyspnea and initial hospitalization on plasma measurements; however, as a result, the effect of cardiac treatment on RAAS biomarkers must be considered. Free plasma cortisol was below the lower limit of quantification in the majority of dogs studied at both timepoints, suggesting that this assay might not be clinically useful in dogs with CHF.

In conclusion, plasma cortisol was not correlated with cardiac or all-cause mortality in our study sample of dogs with CHF secondary to MMVD. Treatment with ACEi and spironolactone were associated with longer survival in the CHF dogs studied on univariate analysis. This association was no longer statistically significant after FDR correction or on multivariate analysis for treatment with ACEi.

## Supplementary Material

Supplemental_Figure_1_aalag063

Supplemental_Figure_1_Caption_aalag063

## Off-label antimicrobial declaration

The authors declare no off-label use of antimicrobials.

## Institutional animal care and use committee or other approval declaration

The authors declare that ethics approval and owner consent was obtained for each dog prior to study enrollment (Iowa State University IACUC protocols 19-213 and 22-106, University of Bristol VIN/20/028).

## Abbreviations

AA2: ratio of aldosterone to angiotensin II
ACE: angiotensin-converting enzyme
ACEi: angiotensin-converting enzyme inhibitor
ACE-S: ratio of angiotensin II to angiotensin I
Ang: angiotensin
BUN: blood urea nitrogen
CHF: congestive heart failure
CRP: C-reactive protein
LA:Ao: left-atrium-to-aorta ratio
LV FS%: left ventricular fractional shortening
LVIDdN: left-ventricular internal diameter in diastole normalized to body-weight
MMVD: myxomatous mitral valve disease
MR: mineralocorticoid receptor
MRB: mineralocorticoid receptor blocker
NT-proBNP: N-terminal pro-B-type natriuretic peptide
PRA-S: sum of angiotensin I and angiotensin II
RAAS: renin–angiotensin–aldosterone system
