# Supplementary figures and images for: Prognostic value of plasma cortisol concentration in dogs with congestive heart failure
Source: J Vet Intern Med. 2026 Apr 8;40(2):aalag063. doi: 10.1093/jvimsj/aalag063 (PMC13069890; doi:10.1093/jvimsj/aalag063)

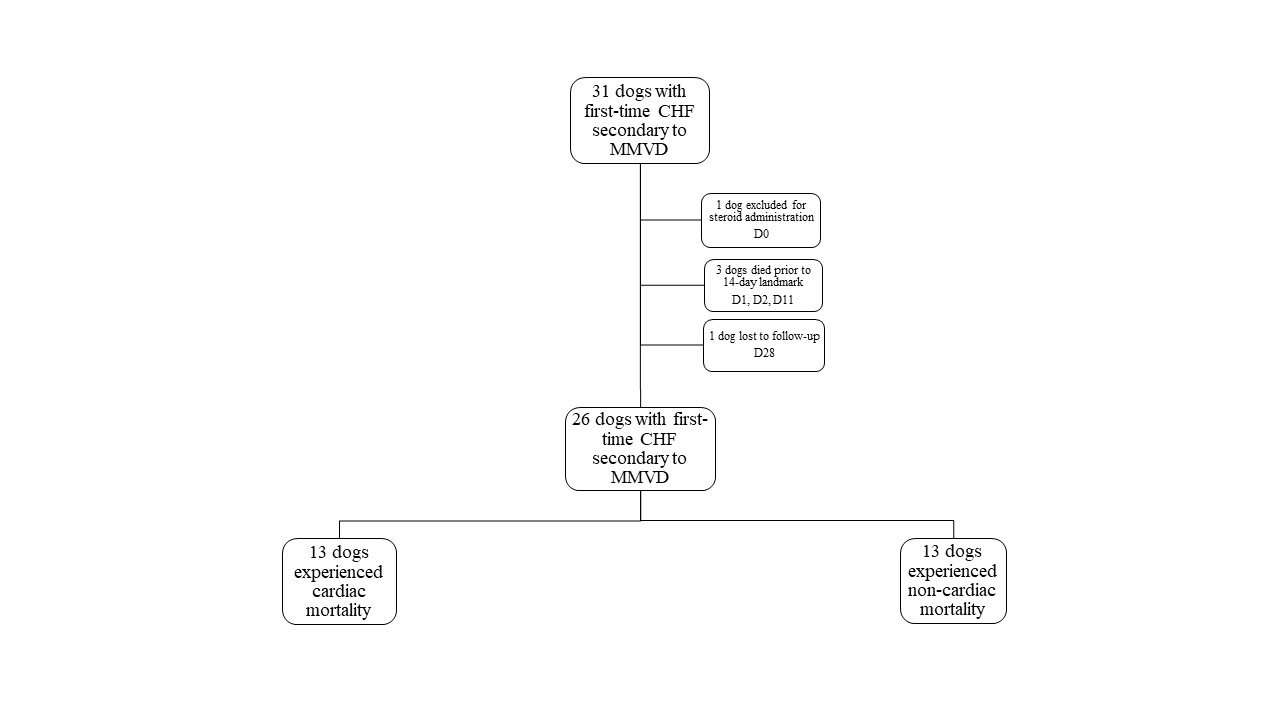

Supplement: Supplemental_Figure_1_aalag063 [file supplemental_figure_1_aalag063.jpeg]
