# Supplementary material for: Prognostic value of plasma cortisol concentration in dogs with congestive heart failure
Source: J Vet Intern Med. 2026 Apr 8;40(2):aalag063. doi: 10.1093/jvimsj/aalag063 (PMC13069890; doi:10.1093/jvimsj/aalag063)
Supplement: Supplemental_Figure_1_Caption_aalag063 [file supplemental_figure_1_caption_aalag063.docx]

Supplemental Figure 1: Flow diagram of the study population of 31 dogs with congestive heart failure secondary to myxomatous mitral valve disease. Date of exclusion after enrollment from the study is listed were relevant. Study enrollment and follow-up occurred between August 2019 – February 2024. CHF, congestive heart failure; MMVD, myxomatous mitral valve disease.
